# Concrete Compressive Strength by Means of Ultrasonic Pulse Velocity and Moduli of Elasticity

**DOI:** 10.3390/ma14227018

**Published:** 2021-11-19

**Authors:** Bogdan Bolborea, Cornelia Baera, Sorin Dan, Aurelian Gruin, Dumitru-Doru Burduhos-Nergis, Vasilica Vasile

**Affiliations:** 1 Civil Engineering Faculty, Politehnica University of Timisoara, 300223 Timisoara, Romania; sorin.dan@upt.ro; 2 NIRD URBAN-INCERC Timişoara Branch, 300223 Timisoara, Romania; cornelia.baera@incd.ro (C.B.); aurelian.gruin@incd.ro (A.G.); 3 Research Center in Engineering and Management, Politehnica University of Timisoara, 300191 Timisoara, Romania; 4 Civil Engineering Faculty, IOSUD-UTCN Doctoral School, Technical University of Cluj-Napoca, 400020 Cluj-Napoca, Romania; 5 Faculty of Materials Science and Engineering, Gheorghe Asachi Technical University, 700050 Iasi, Romania; 6 NIRD URBAN-INCERC Bucharest Branch, 021652 Bucharest, Romania; valivasile67@yahoo.com

**Keywords:** concrete, compressive strength, NDT, ultrasonic pulse velocity, modulus of elasticity

## Abstract

Developing non-destructive methods (NDT) that can deliver faster and more accurate results is an objective pursued by many researchers. The purpose of this paper is to present a new approach in predicting the concrete compressive strength through means of ultrasonic testing for non-destructive determination of the dynamic and static modulus of elasticity. For this study, the dynamic Poisson’s coefficient was assigned values provided by technical literature. Using ultra-sonic pulse velocity (UPV) the apparent density and the dynamic modulus of elasticity were determined. The viability of the theoretical approach proposed by Salman, used for the air-dry density determination (predicted density), was experimentally confirmed (measured density). The calculated accuracy of the Salman method ranged between 98 and 99% for all the four groups of specimens used in the study. Furthermore, the static modulus of elasticity was deducted through a linear relationship between the two moduli of elasticity. Finally, the concrete compressive strength was mathematically determined by using the previously mentioned parameters. The accuracy of the proposed method for concrete compressive strength assessment ranged between 92 and 94%. The precision was established with respect to the destructive testing of concrete cores. For this research, the experimental part was performed on concrete cores extracted from different elements of different structures and divided into four distinct groups. The high rate of accuracy in predicting the concrete compressive strength, provided by this study, exceeds 90% with respect to the reference, and makes this method suitable for further investigations related to both the optimization of the procedure and = the domain of applicability (in terms of structural aspects and concrete mix design, environmental conditions, etc.).

## 1. Introduction

Non-destructive testing methods (NDT) are essential tools in estimating concrete properties (mechanical or physical). A comprehensive analysis of the mechanical properties is useful in the process of structural optimization, as well as in terms of budget efficiency.

In the case of Reinforced Concrete (RC) structures, one of the key properties is the compressive strength. An investigation from this point of view can provide an overview of the structural integrity of a building. Such an analysis helps civil engineers in optimizing the process of structural intervention by deepening the understanding of how the building works from the structural point of view and also considering the concrete mix design and the associated physical, mechanical, and durability characteristics. Therefore, the interventions can be targeted on those elements that have a deficient behavior, which can induce negative effects into the structure [1,2,3,4,5,6,7].

Traditionally, the concrete compressive strength is determined through destructive testing (DT) which is considered the most reliable testing, and thus been referred to as the reference method. In DT, there are identified three possible situations:

- The samples are prepared and tested in the laboratory;
- The samples are collected on the construction site during the concrete casting, followed by curing and testing in the laboratory;
- The concrete cores are extracted directly from the structure (specific elements) and, after specific processing and conditioning, they are tested in the laboratory.

In the first situation, this testing is performed in order to evaluate or calibrate different mixing sequences of concrete mixture [8,9]. The samples collected on the construction site during the concrete casting are usually considered in cases of new buildings, to assess and confirm the concrete class [10] or to check compressive strength at intermediate specific terms (identity tests at 1 day, 2 days, 7 days, etc.). In the case of existing buildings, DT is performed by extracting concrete cores from certain concrete elements, such as columns, beams, slabs, diaphragms, etc., and then subjecting them to a compressive load until failure [11]. The preparation of the cores is made with respect to specific regulations and procedures in order to ensure the necessary testing accuracy [12].

According to [13,14] the analysis procedure depends on the amount of available information regarding the existing structure to be evaluated: information about the construction geometry, elements detailing, and material type determine the appropriate Knowledge Level (KL) of the structure under study. There are considered to be three KLs, defined as follows: KL1—limited, KL2—normal, and KL3—full, and to each of them is assigned a Confidence Factor (CF): (CF_KL1_ = 1.35; CF_KL2_ = 1.20; and CF_KL3_ = 1.00). The confidence factor is used as a correction factor for incomplete knowledge and level of uncertainty [11]. To reach a superior level of confidence (KL3 level, for instance), a large number of cores must be extracted from the structure, which can cause several inconveniences such as they can be time- and resource-consuming and also affect the structure itself due to specimens’ extraction. Furthermore, the compressive strength may vary within the same element, due to the specific heterogeneity of concrete [15].

NDT represents a possible, viable alternative, mainly in terms of cost efficiency and also as they are fast in delivering results. However, NDT techniques measure indicators that are sensitive to a specific concrete property. For example, the ultrasonic pulse velocity and rebound hammer are sensitive to mechanical properties such as the compressive strength and porosity of concrete [16]. Another major problem pointed out by Angst [17] is the fact that the relation between mechanical properties and measured indicators is not constant. This is attributed to several causes, strongly connected to concrete physical characteristics (its specific heterogeneity, the porosity, water content, aggregate maximum dimension, etc.) and also to element exposure, measured data accuracy, and limited number of measurements. 

Over the years, several NDT methods have been developed with the main purpose of estimating, as correctly as possible, the mechanical properties of materials and elements. A short overview of the NDT methods used on concrete structures is presented thus:

- One of the first and most used NDTs is the visual inspection (VI) [18]. It focuses on identifying visible pathologies in concrete such as cracks, voids, and spalling and trying to understand what caused them. It is a subjective and limited method, as internal characteristics of the structure cannot be determined, but it is also an important first step for further evaluation, providing useful information for establishing optimum and adequate methodologies (DT or NDT) for further investigation.
- The rebound hammer (RHS) determines the surface hardness. The main advantages are the fact that is a simple to use method with low cost and energy. The method tests the concrete strength on a depth of 2–3 cm, this being the reason why it should be combined with other methods that tests the concrete elements in depth [19].
- The radiographic testing (RT) consists of a radioactive isotope source that transmits photons continuously through the concrete element, photons which are developed on a radiation sensitive film. It is mainly used for visualizing interior features of an element. There are many types of radiography that each have a specific application. In order to detect voids, cracks, or other interior defects, gamma-ray radiography was found to be useful [20]. This method is rarely used due to safety concerns.
- The carbonatation testing (CT) is made by spraying an exposed surface of the concrete element with a solution containing 1% phenolphthalein. The calcium hydroxide reacts with the solution resulting in a pink color, while the carbonated area will remain uncolored [21].
- Infrared thermography (IT) is used in order to determine the internal voids, cracks, or delamination by measuring the time delay before the temperature changes [22].
- The electromagnetic and radar testing (E&RT) are the most used NDT methods for the identification of reinforcements position, diameter, and distance from the surface [23].
- The drilling resistance method (DRM) presumes estimating the concrete compressive strength by counting the time required to drill a certain distance in the concrete element with a constant force and rotation speed. Serkan et al. [24] proved the accuracy of the method and presented good results when it was combined with the rebound hammer testing.
- Ultrasonic pulse velocity (UPV) is an NDT which has been extensively investigated for decades [25]. The method consists of measuring the transit time of an ultrasonic pulse from a transmitter to a receiver, knowing the distance between the two transducers. A short amount of time is needed for the ultrasonic pulse to pass through an element result in a high velocity, meaning a compact and homogenous material. This is an indication of the element’s strength.

The most used NDT methods, for estimating concrete compressive strength, are the Schmidt rebound hammer, the ultrasonic pulse velocity, and the sonic rebound (SonReb) which consists of a combination of the first two methods. Făcăoaru et al. [26] developed and described the procedure which consists of applying some correctional factors based on cement type and dosage, granulometry and type of aggregates, and concrete age. SonReb has a high degree of efficiency and is still used worldwide, successfully, in estimating concrete compressive strength. Still, one of the most important disadvantages of the method is its requirements of mix design information regarding the evaluated concrete; in the case of older structures this information is not always available, which may lead to unprecise results. The viability of the method should also be verified on various types of concrete mix design developed with different additions, waste, or by-products (mineral, rubber, plastic, glass, etc.), which gained large diversity in recent years due to environmental protection requirements and Circular Economy implementation [2,3,4].

Researchers tried to develop various relationships between the measured indicators and the mechanical properties of concrete, by using different techniques, such as response surface (RS) [27,28,29,30,31,32,33,34], data fusion (DF) [35,36,37], and artificial neural networks (ANN) [38,39,40,41,42,43]. The empirical relationships developed over the years have a different structure: linear (LN) [40,41], polynomial (PL) [44,45], and power (PW) [46,47]. Sbartai et al. [34] report a satisfactory level in predicting concrete properties based on ultrasonic pulse velocity, ground penetration radar (GPR), electrical resistivity measurements, and data interpretation through the means of the response surface. However, when the data is interpreted with the help of ANN, the results have a higher rate of predictability. Asteris et al. [40] developed and optimized an ANN that considers the ultrasonic pulse velocity and Schmidt rebound hammer as the input values needed in order to estimate the concrete compressive strength. Based on the statistical parameters employed to evaluate the performance, the developed ANN model proved to have high efficiency in estimating the compressive strength, both when applied on its own database and also applied on other databases of different researchers. Khademi et al. [41] compared different techniques used to predict the 28 days compressive strength of concrete. In their mentioned study, a multiple linear regression (MLR), an artificial neural network, and adaptive neuro-fuzzy inference system models (ANFIS) were implemented with the purpose of finding the most accurate method of estimating concrete compressive strength. It was concluded that both ANN and ANFIS models can predict the concrete compressive strength more accurately than MLR, which proved to be unreliable. This is due to the fact that these models consider the non-linear correlation between the variables used as input data. It was also concluded that the accuracy of prediction is influenced by the number of input variables.

Breysse concluded [16] that a universal law between NDT and concrete compressive strength does not exist, despite the fact that many authors tried to find one.

This paper aims to present a methodology in estimating the concrete compressive strength by using ultrasonic pulse velocity as the only on-site testing method and a series of mathematical relations connecting the UPV with the moduli of elasticity (dynamic and static) and finally with the compressive strength.

## 2. Materials and Methods

### 2.1. The Destructive Method (DT)

This method is considered to deliver the most reliable results regarding the concrete compressive strength and, in this study, all results are reported to this method, considered to be the reference one. Consequently, the precision rate of the proposed method is also established with respect to the DT, as reference base of evaluation. DT consists of extracting concrete cores from the existing elements, cores which are then subjected to a series of laboratory processing and conditioning after which they are subjected to compression load until failure. The resulting compressive bearing capacity (f_car_) is corrected by a series of coefficients described in Equation (1) provided by Romanian Norm NP 137 [12], thus resulting in the equivalent concrete compressive strength (f_is_).

(1) $$f_{\mathrm{is}}=a\cdot b\cdot c\cdot e\cdot g\cdot d\cdot f_{\mathrm{car}}$$

where: f_is_—equivalent concrete compressive strength (MPa); a—coefficient that takes into account the influence of the core diameter; b—coefficient that takes into account the height/diameter ratio; c—coefficient that takes into account the influence of the degraded layer; e—coefficient that takes into account the nature of the leveling layer; g—coefficient that takes into account the humidity of the concrete core; d—coefficient that takes into account the position and diameter of the reinforcement bars; and f_car_—resulted compressive bearing capacity (MPa).

As mentioned in the previous paragraph, destructive testing inflicts damage on the tested element; therefore, the number of cores must be maintained to a minimum in order to preserve the structural integrity of the element. For this reason, it is possible that the obtained values, calculated on an insufficient number of specimens, namely extracted cores from a designated element or assembly, do not reflect the overall value of the compressive strength of the targeted element. Additionally, in some cases, the extraction of the concrete core itself can prove to be difficult or even impossible to perform due to technological conditions such as the position of the designated element in the structure, the possibility to fix the drilling machine in order to extract the concrete core, etc.

### 2.2. Ultrasonic Pulse Velocity (UPV)

The ultrasonic pulse velocity was used as the on-site NDT testing method. In accordance with the theory of sound propagation in solids, the velocity of the ultrasonic signal depends on the density and elastic modulus of the material subjected to testing [48]. 

A calibration between compressive strength and ultrasonic pulse velocity for each concrete sample assures enough dependability for the two indicators [49]. Naik et al. [50] presented a full review of the method. ultrasonic pulse velocity can be determined with Equation (2) presented by Romanian Norm NP 137 [12].

V_L_ = L/T (2)

where: V_L_—ultrasonic pulse velocity (km/s); L—path length in concrete (mm); and T—transit time (µs).

### 2.3. Theoretical Considerations

Modulus of elasticity of concrete (E) is a property of concrete that estimates the potential deformation of a structural element under service conditions [51]. The factors influencing this property are the dosage of cement, concrete age and class, the binder characteristics, and proportions.

The static modulus of elasticity (E_s_) is a fundamental parameter that is defined by the stress–strain diagram under static loads [51] and it is generally estimated based on design code, not on direct measurements.

The dynamic modulus of elasticity (E_d_), in comparison to E_s_, is defined by the ratio of stress–strain under vibratory conditions [52]. The most common techniques for determining E_d_ are resonance frequency or UPV [53], but a study conducted by Luo and Bungey [54] presented a new approach by using surface waves in order to determine E_d_. For this study, E_d_ was determined accordingly to Romanian Guide GE 039 [55] via UPV using Equation (3).

(3) $$E_{d}=\frac{\left(1+\Theta_{d}\right)\cdot \left(1-2\cdot \Theta_{d}\right)}{1-\Theta_{d}}\cdot \frac{\gamma }{g}\cdot V_{L}^{2}$$

where: E_d_—dynamic modulus of elasticity (MPa); Θ_d_—dynamic Poisson’s ratio; γ—air dry density (kg/m^3^); g—gravitational acceleration (m/s^2^); and V_L_—ultrasonic pulse velocity (km/s).

Romanian Guide GE 039 [55] presents a mathematical expression, Equation (4), for the determination of the dynamic Poisson’s ratio, but for this study, the dynamic modulus of elasticity was assumed the value presented by the technical literature [55], namely Θ_d_ = 0.25 (for concrete preserved in the air).

(4) $$\Theta_{d}=\frac{{\left(2\cdot n\cdot l\right)}^{2}}{V_{L}^{2}}$$

where: n—fundamental resonant frequency (cycles/sec); l—length of specimen (m); and V_L_—ultrasonic pulse velocity (km/s).

Thereby, when considering the Θ_d_ = 0.25 the values of the function depending on the dynamic Poisson’s ratio becomes:

(5) $$f\left(\Theta_{d}\right)=\frac{\left(1+\Theta_{d}\right)\cdot \left(1-2\cdot \Theta_{d}\right)}{1-\Theta_{d}}=0.83$$

Inserting Equation (5) in Equation (3) results the dynamic modulus of elasticity has the following expression:

(6) $$E_{d}=0.83\cdot \frac{\gamma }{g}\cdot V_{L}^{2}$$

Regarding the air-dry density Salman [56] and Panzera et al. [57] conducted studies to find a linear correlation between air-dry density (γ) and UPV. In this study, Equation (7), presented by Salman [56] was used to determine the air-dry density of concrete.

(7) $$\gamma =114.8\cdot V_{L}+1813$$

where: γ—air-dry density (kg/m^3^) and V_L_—ultrasonic pulse velocity (km/s).

In order to establish the accuracy of the proposed equation, the air-dry density of concrete was experimentally determined. The samples were weighed and measured with the purpose of determining the apparent volume. Comparing the mean values of air-dry density obtained experimentally (γ_e_) with the mean values of the predicted ones using Equation (7) (γ_t_), it was shown it reached a precision rate of 98%.

Furthermore, the theoretical air-dry density was used in this study as it was proven to be efficient, thus the method remained completely non-destructive and depended only on UPV.

Romanian Guide GE 039 [55] stipulates that the ratio between E_s_ and E_d_ ranges, in general, between 0.85–0.95. For this study, the correlation between the two moduli of elasticity was determined by experimentally. Therefore, each modulus of elasticity (E_s_ and E_d_) was calculated individually and then a direct link between them was established. E_d_ was determined via UPV (Equation (6)) and E_s_ was determined via DT (Equation (8)).

For determining the static modulus of elasticity, with the air-dry density determined with Equation (7) and compressive strength obtained destructively (f_is_) determined with Equation (1), using the mathematical relationship presented by Noguchi et al. [51] (Equation (8)), a static modulus of elasticity could be determined.

(8) $$E_{s}=2.1\cdot 10^{5}\cdot {\left(\frac{\gamma }{2.3}\right)}^{1.5}\cdot {\left(f_{c}/200\right)}^{1/2}$$

where: E_s_—static modulus of elasticity (MPa); f_c_ = f_is_—concrete strength (MPa); and γ = γ_t_—concrete air-dry density determined via UPV (kg/m^3^).

The dynamic modulus of elasticity was mathematically calculated with Equation (6), using the ultrasonic pulse velocity. 

Comparing the values of the two moduli of elasticity, determined for each specimen separately, it was established a direct and linear link between them described in Equation (9).

(9) $$E_{s}=0.75\cdot E_{d}$$

Using Equation (9), the static modulus of elasticity can now be determined only from the ultrasonic pulse velocity measurements and using Equation (7) the air-dry density can be obtained through the same measurements. Therefore, in Equation (8) the only unknown parameter remains concrete compressive strength (f_c_). Extracting that parameter and rewriting Equation (8) results in a relationship (Equation (10)) where the compressive strength value depends only on parameters that can be determined via UPV.

(10) $$f_{c}=(E_{s}^{2}\cdot 200)\cdot {\left[2.1\cdot 10^{5}\cdot {\left(\gamma /2.3\right)}^{1.5}\right]}^{2}$$

### 2.4. Experimental Procedure

**Figure 1:**
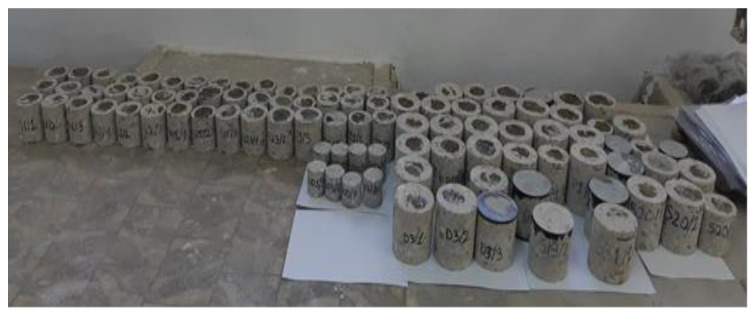
Concrete core specimens.

The study was conducted on 90 concrete cores with a diameter of 74 and 94 mm, extracted from different elements of different structures (Figure 1). The elements include the raft foundation, columns, beams, and reinforced concrete walls. After the extractions of the cores, the processing was conducted in accordance with Romanian Norm NP 137 [12]. The specimens were cut at both ends with a wet diamond disk and then dry air stored in laboratory conditions T: (21 ± 3) °C and RH: (50 ± 5)%, in accordance to Romanian Norm NP 137 [12]. The core specimens were cured for five days before weighting and UPV testing. This conditioning was performed to avoid that the humidity resulting from the wet cutting would affect the UPV data. Figure 1 presents the concrete sample after specific cutting and conditioning and before the destructive testing.

**Figure 2:**
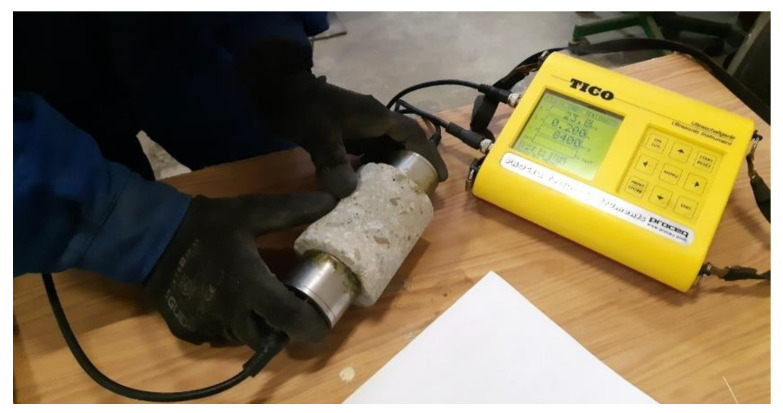
UPV testing.

The as-received state density was determined with respect to SR EN 12390-7 [58] by specimens air-dry curing and then their measuring (diameter and height, for volume calculation), followed by their weighting. Then, the specimens were tested via UPV with a Tico Proceq device equipped with 54 kHz transducers (Figure 2). The coupling agent for the transducers was Vaseline. The last step was testing destructively the specimens, in compression. This procedure was conducted with respect to SR EN 12390-3 [59] which stipulates the curing and the testing conditions: the recommended dimensions of the concrete cores, the air-dry exposure, the preparation, and positioning, etc., as well as the loading rate. The destructive testing was conducted with a hydraulic press at a loading rate of 0.6 MPa/s.

**Figure 3:**
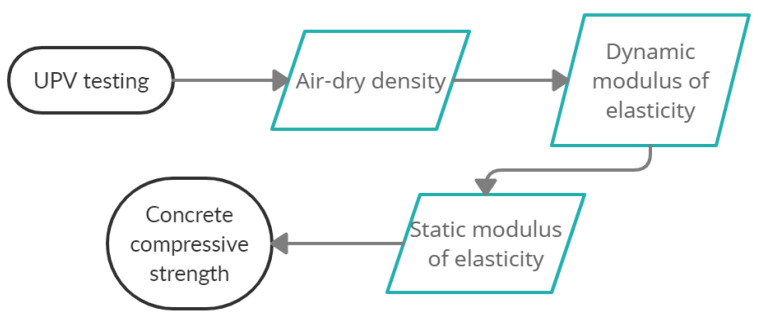
Flowchart of the presented method.

Figure 3 presents the flowchart of the proposed method, consisting of the sequence of the major considered steps, in terms of experimental testing (black curve contour) and the corresponding parameters (light blue contour) determined by using the previously collected data. 

## 3. Results and Discussions

### 3.1. Proposed Method Compared to DT

For a more accessible interpretation of the results, the core specimens were divided into four groups. The considered division criterion is the value of compressive strength (f_is_) determined via the Destructive Method, as follows:

- Group 1 [15–20) MPa: f_is_ ranges from 15 to 20 Mpa;
- Group 2 [20–25) Mpa: f_is_ ranges from 20 to 25 Mpa;
- Group 3 [25–30) Mpa: f_is_ ranges from 25 to 30 Mpa;
- Group 4 ≥ 30 MPa: f_is_ exceeds 30 MPa.

The theoretical methods for assessing the concrete air-dry density and compressive strength, for the four considered groups of specimens, were statistically evaluated with respect to the experimental and reference procedures, in terms of coefficient of variation (CoV), and also the accuracy (A_c_).

CoV is defined by Everitt [60] by the means of Equation (11), as the ratio between the standard deviation (σ) and the mean value (µ) of the group of specimens where applied.

(11) $$\mathrm{CoV}=\frac{\sigma }{\mu }$$

where: CoV—coefficient of variation (%); σ—standard deviation; and μ—mean value.

The air-dry density and compressive strength (measured and predicted) were also analyzed in terms of accuracy, defined in accordance with ISO 5725-1 [61] as the ratio between the predicted value (result of the proposed method) and the “true” value, provided by the reference method. Accuracy was calculated by the use of Equation (12).

(12) $$A_{c}=\frac{\mathrm{Predicted}\;\mathrm{value}}{\mathrm{Measured}\;\mathrm{value}}\cdot 100$$

where: A_c_—accuracy (%).

Table 1 presents the air-dry density values for each of the four specimen groups, determined by using both methods: the experimental method, (comprises specimens’ measurement and weighing) and the Salman theoretical method (based on UPV individual values) [56]. The accuracy was calculated by using as input data the mean values recorded for each of the four groups of core specimens.

**Figure 4:**
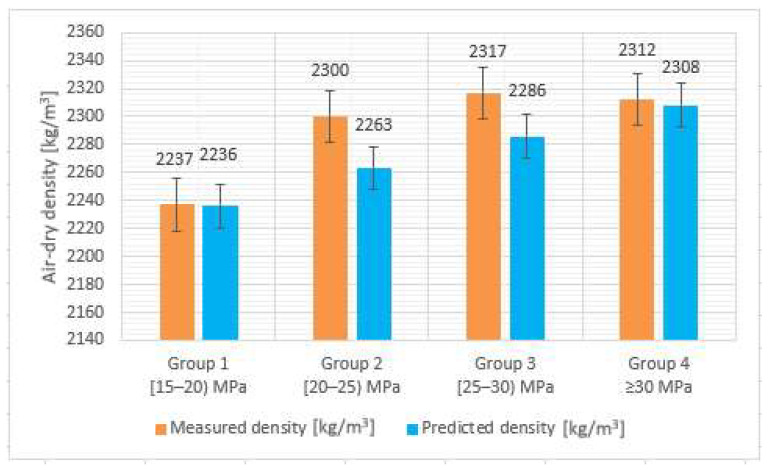
Air-dry density, mean values.

**Figure 5:**
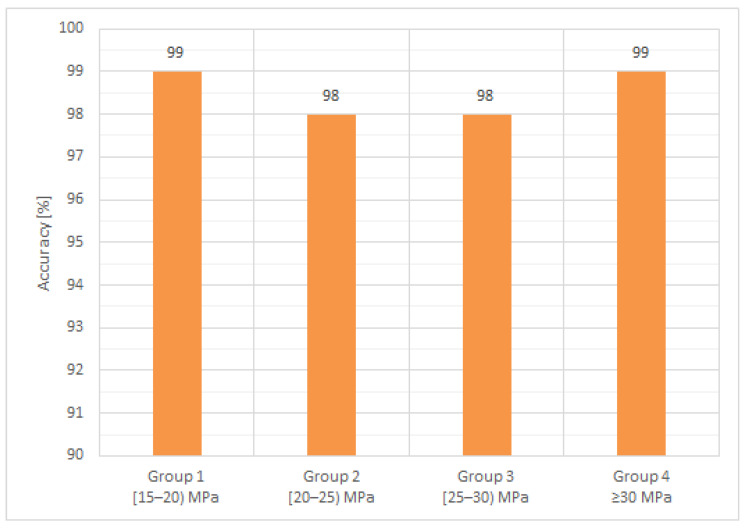
Air-dry density, the accuracy of the NDT method with respect to the reference.

Figure 4 and Figure 5 present a graphical representation of the mean values and accuracy of measured and predicted density. With an accuracy ranging between 98% and 99%, the theoretical Salman method [56] for determining the density via UPV testing proves to be a viable approach. This conclusion is also supported by the CoV values, ranging from 1.3% to 2.7% for the reference method (experimental measurements) and presenting a more compact range of smaller CoV values, from 0.4% to 0.8%, for the theoretical, Salman approach.

Table 2 presents the results of compressive strength for each of the four core groups, determined by both methods: the proposed method (UPV testing and interpretation via moduli of elasticity) and the reference, destructive testing.

**Figure 6:**
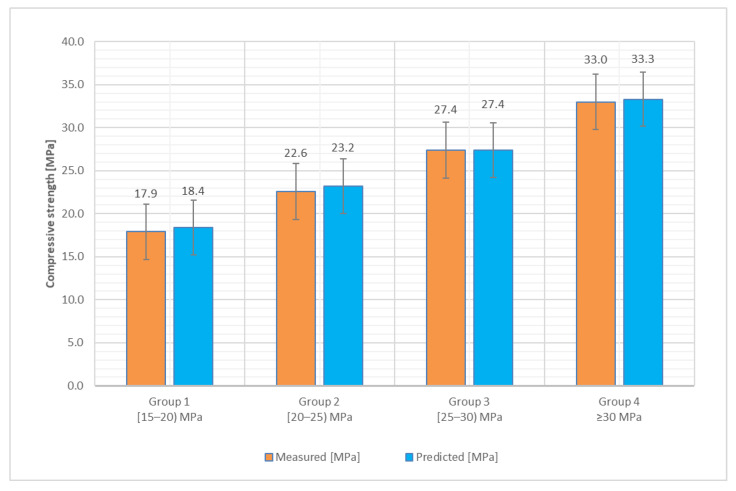
Compressive strength, mean values.

**Figure 7:**
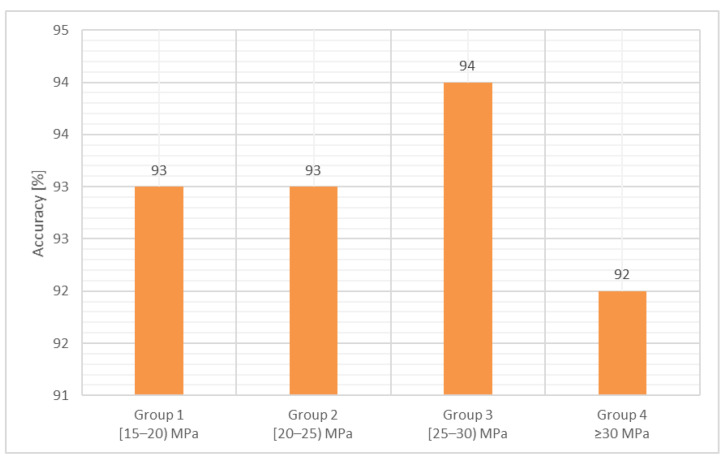
Compressive strength, the accuracy of the proposed method related to the reference.

In terms of compressive strength, which is the main focus of the study, a graphical representation of the mean values and accuracy for each of the four groups is presented in Figure 6 and Figure 7. The specimens sorting into four distinct groups function of the compressive strength, as previously specified, was considered proper for a better understanding of the phenomenon and to facilitate the data processing and the scattering of results. The wide range of values for the DT compressive strength (f_is_) can be noted, with a minimum of 15.8 MPa and a maximum of 38.9 MPa. Additionally, the corresponding values for the NDT testing (f_c_) range from 15.1 MPa to 37.0 MPa (Table 2). 

The accuracy of the proposed method for compressive strength determination via UPV and moduli of elasticity was also calculated using Equation (12), with respect to the mean values for each of the four groups of core specimens, presented in Table 2. The obtained results proved to be satisfactory, as the variation is very small with respect to the DT, regardless of the wide range of values. This conclusion is also supported by the CoV values (Table 2), ranging from 4.7 to 6.8% for the reference method and offering a similar compact range of values from 7.7 to 10% for the proposed method.

Furthermore, when analyzing each group, it can be noticed that for groups 1, 2, and 4, the theoretical compressive strength, evaluated via the proposed method, tends to be a little overestimated with respect to the reference (2.8, 2.7 and 0.9%, respectively), while in the third group there is a clear match of the values.

Figure 7 graphically presents the accuracy evaluation, in terms of mean values. It can be noticed that the values are ranging from 93 to 94%.

As presented in Figure 7, the precision of this method in terms of Ac reaches 93% for the first two groups, 94% for the third group, and 92% for the fourth group. Considering all the values, the mean value of the precision is up to 93%.

**Figure 8:**
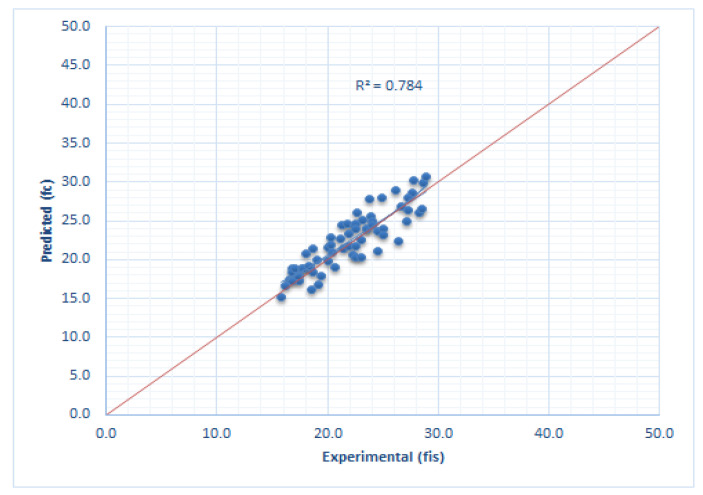
Correlation between experimental and calculated compressive strength.

Figure 8 presents a graphical representation of the correlation between experimentally determined compressive strength (f_is_) and predicted compressive strength (f_c_) is presented.

A good correlation is achieved between the two sets of values. The coefficient of determination r^2^, which is a statistical indicator of the quality of the theoretical model, is in this case equal to 0.78.

### 3.2. Proposed Method and SonReb Method Compared to Destructive Method

For a better validation of the proposed method, the obtained results are compared to both the SonReb method and the DT. For the SonReb method, six structural elements (columns) were investigated on-site by using ultrasonic pulse velocity and Rebound Hammer Schmidt. Each element was tested in three sections, with five UPV and nine RHS measurements/section. In Table 3, the resulting mean values of the UPV, RHS, and compressive strength from each method are presented.

**Figure 9:**
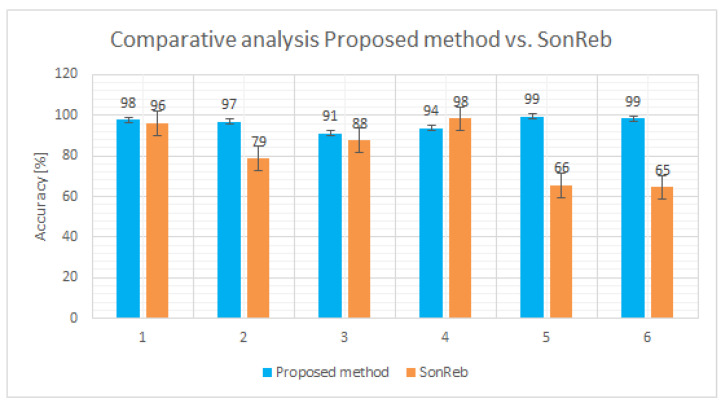
Graphical representation of the accuracy evaluation of each method, proposed and SonReb method, with respect to the reference method (destructive testing).

A graphical representation of the accuracy is presented in Figure 9, namely a comparative analysis between the proposed method vs. SonReb method, both of them evaluated with respect to the reference, namely, the destructive method.

For the six elements investigated through both NDT methods, the precision rate in the case of the proposed method reaches up to 96%, while in the case of the SonReb method the precision reaches up to 82%. 

## 4. Conclusions

The aim of this paper is to present the results obtained by combining the on-site measurements of UPV and theoretical interpretation using a set of equations developed by different researchers linking the values of ultrasonic pulse velocity to the dynamic modulus of elasticity, static modulus of elasticity, and finally concrete compressive strength.

Estimating concrete compressive strength through this method delivered results with high accuracy, which, in this case, ranged between 84 and 100%. It can be noticed that the high level of accuracy remains the same regardless of the range value of compressive strength, which in this study is between 15.8 and 38.9 MPa. Additionally, the coefficient of variation (CoV) shows reduced values, ranging along compact intervals, both for the dry-air density evaluation (from 0.4 to 0.8%, for the theoretical, Salman approach) and also for the compressive strength evaluation (from 7.7 to 10%, for proposed method). Further investigations will also consider the methodology and statistical approach proposed by Breysse et al. [62].

As this method relies only on UPV measurements, the on-site surface preparation and testing process must be performed with a high level of precision; otherwise, the results will have a higher level of uncertainty.

In contrast to the SonReb method, the proposed one has the advantage, so far, that there is no requirement of information about the classical concrete mix design such as cement type and dosage, granulometry, and nature of aggregates. This information is often difficult to obtain, especially in the case of buildings where the concrete mix was produced on-site with no known recipe. In the SonReb method, not knowing these parameters correct can lead to errors up to ±30%. In this case, although the concrete mix design was known, hence all the coefficients were correctly assumed, the accuracy of the SonReb method had a lower value than the proposed method on each analyzed concrete column when compared to DT.

The current results are clearly encouraging, offering new research perspective for method optimization and further confirmation to prove its viability, especially in terms of concrete mix design diversity, which has experienced an exponential growth in the last decades. The single and multiple additions in concrete compositions, waste, or by-products generated by the industry, may induce concrete hardened state changes which lead to complex investigation in terms of overall behavior, NDT included. A preliminary approach on this area represents the on-going study of the current research. 

## Figures and Tables

**Table 1:** Air-dry density.

Density (kg/m^3^)									
	Measured Density (γ_e_)				Predicted Density (γ_t_)				Accuracy (%)
	Min	Mean	Max	CoV (%)	Min	Mean	Max	CoV (%)	
Group 1 [15–20) MPa	2149	2237	2319	1.7	2215	2236	2254	0.4	99
Group 2 [20–25) MPa	2210	2300	2384	2.1	2241	2263	2286	0.5	98
Group 3 [25–30) MPa	2221	2317	2411	2.7	2259	2286	2347	0.8	98
Group 4 ≥30 MPa	2256	2312	2365	1.3	2281	2308	2322	0.6	99

**Table 2:** Compressive strength.

Compressive Strength (MPa)									
	DT Compressive Strength (f_is_)				NDT Compressive Strength (f_c_)				Accuracy (%)
	Min	Mean	Max	CoV (%)	Min	Mean	Max	CoV (%)	
Group 1 [15–20) MPa	15.8	17.9	20.0	6.8	15.1	18.4	21.6	8.9	94
Group 2 [20–25) MPa	18.3	22.6	25.0	7.1	19.1	23.2	27.9	9.6	93
Group 3 [25–30) MPa	25.1	27.4	29.6	4.7	22.4	27.4	30.7	7.7	94
Group 4 ≥30 MPa	30.2	33.0	38.9	6.3	26.9	33.3	37.0	10	94

**Table 3:** The results obtained from each presented method.

Element	Mean UPV (km/s)	Mean RHS (div)	Mean Concrete Compressive Strength		
			Proposed Method	SonReb Method	Destructive Method
Column 1	4.324	40	26.1	25.7	26.7
Column 2	4.142	37	28.6	21.8	27.7
Column 3	3.985	38	24.7	19.7	22.5
Column 4	3.716	41	18.9	17.4	17.7
Column 5	3.632	35	17.3	11.4	17.4
Column 6	3.758	35	19.7	12.9	20.0
